# Signal peptide replacement resulted in recombinant homologous expression of laccase Lcc8 in *Coprinopsis cinerea*

**DOI:** 10.1186/s13568-019-0761-1

**Published:** 2019-03-15

**Authors:** Marcus Schulze, Lukas Geisler, Andrzej Majcherczyk, Martin Rühl

**Affiliations:** 1 Institute of Food Chemistry and Food Biotechnology, Justus Liebig University Giessen, Heinrich-Buff-Ring 17, 35392 Giessen, Germany; 2 Molecular Wood Biotechnology and Technical Mycology, Büsgen-Institute, University of Goettingen, Büsgenweg 2, 37077 Goettingen, Germany

**Keywords:** Laccase, *Coprinopsis* *cinerea*, Signal peptide, Multi-copper oxidase, Purification, Biochemical characterization

## Abstract

Although the model agaricomycete *Coprinopsis cinerea* possess 17 different laccase genes, up to now only four *C. cinerea* laccases have been purified and characterized to some degree. By exchanging the nucleotide sequence of the deduced signal peptide of Lcc8 it was possible to homologously express *lcc8* in *C.* *cinerea* under control of the *Agaricus bisporus gdpII* promoter and the *C.* *cinerea lcc1* terminator. The purified Lcc8 showed two bands in the SDS-PAGE with a molecular weight of 64 kDa and 77 kDa, respectively. The IEF determined pI values of 3.3 and 3.4 for both bands. The optimal pH for oxidation of the substrates ABTS, 2,6-dimethoxyphenol, guaiacol and syringaldazine was pH 4.0, pH 5.0, pH 4.5 and pH 5.0, respectively. Best pH for enzyme storage was pH 8.0. The optimal temperature for oxidation of ABTS was 63 °C, while Lcc8 showed activity of at least 50% over 300 min at 50 °C. The comparable high stability of Lcc8 at alkaline pH and higher temperatures can be of interest for biotechnical applications.

**Electronic supplementary material:**

The online version of this article (10.1186/s13568-019-0761-1) contains supplementary material, which is available to authorized users.

## Introduction

Laccases (EC 1.10.3.2, benzenediol: oxygen oxidoreductase) are phenoloxidases capable of oxidizing phenolic and aromatic compounds (Leonowicz et al. 2001). In nature, they are widespread in the fungal kingdom particularly in the phylum Basidiomycota, but they also occur in plants, insects and bacteria (Claus 2004). Since decades, fungal laccases are extensively studied and especially laccases of the fungal phylum Basidiomycota are in focus of research with respect to their production, biochemical characteristics and potential biotechnological use (Baldrian 2006; Giardina et al. 2010). However, up to now only a few applications are commercialized, amongst other reasons likely due to the generally low production yields of active enzymes. Supplementing fungal cultures with a wide range of aromatic alcohols and acids can led to elevated laccase yields (Leonowicz et al. 2001; de Souza et al. 2004). Even a fungus/fungus co-culture was able to increase the native laccase activity in the culture supernatant (Hu et al. 2018). Recombinant expression of laccases for the purpose of high enzyme titers is normally attempted in heterologous ascomycetous hosts, such as yeasts, *Aspergillus* species or other filamentous ascomycetes (Mate and Alcalde 2015; Hong et al. 2002; Larrondo et al. 2003; Kiiskinen et al. 2004; Hong et al. 2007). However, a very high titer of recombinant enzyme has been achieved with the homologous expression of the *lac1* gene in the basidiomycete *Pycnoporus cinnabarinus* (Alves et al. 2004) with a laccase yield of 1.2 g L^−1^. Unfortunately, research on basidiomycetous expression systems for industrial relevant enzymes is spare and might be underestimated, resulting in limited studies on recombinant laccase production in fungi of the phylum Basidiomycota.

In higher fungi, laccase genes often occur in larger families (Hoegger et al. 2006; Kües and Rühl 2011) resulting in different isoenzymes (Bollag and Leonowicz 1984; Rühl et al. 2013). The genome of the model organism *C. cinerea* contains 17 laccase genes (Hoegger et al. 2004), but only a handful of derived isoenzymes have been detected in cultures of various *C. cinerea* strains. In submerged cultures of *C. cinerea* monokaryons, amongst them FA2222, laccases Lcc1 and Lcc5 are the main isoenzymes, whereas Lcc2, Lcc9 and Lcc10 were only present sporadically (Rühl et al. 2013). In the *C. cinerea* monokaryon Okayama 7, co-culture with the Mucoromycete *Gongronella* sp. w5 was able to induce Lcc9 as major enzyme (Pan et al. 2014; Hu et al. 2018), leading to the assumption that Lcc9 possibly takes part in the defense response of *C. cinerea*. Beside these native *C.* *cinerea* laccases, several studies focused on their recombinant production for functional studies (Schneider et al. 1999). Nevertheless, characteristics of only four laccases (Lcc1, Lcc2, Lcc6 and Lcc9) have been published (Schneider et al. 1999; Pan et al. 2014; Tian et al. 2014; Wang et al. 2014). In this study, we were able to express the yet uncharacterized Lcc8 of *C. cinerea* homologously by exchanging the native signal peptide of Lcc8.

## Materials and methods

Chemicals were purchased from Sigma-Aldrich (Steinheim, Germany), Amresco (VWR, Germany), Biozyme (Oldendorf, Germany), AppliChem (Darmstadt, Germany), Merck (Darmstadt, Germany). PCR primers were ordered from Eurofins MWG Operon (Ebersberg, Germany) or Biomers (Ulm/Donau, Germany).

### Strains, culture media and culture conditions

*Escherichia coli* strain JM109 was used for propagation of plasmid DNA. It was cultivated in Lennox-LB media (Carl Roth, Karlsruhe, Germany) supplemented with 50 µg L^−1^ ampicillin (Carl Roth) at 37 °C and 180 rpm. *Saccharomyces cerevisiae* strain RH1385 (*MATa∆ura3*, Mösch et al. 1990) was used for homologous recombination of expression plasmids (Gietz and Schiestl 2007) as described previously (Galperin et al. 2016). *Coprinopsis cinerea* strain FA2222 (DSM 28333) was used as the expression host. Cultivation on agar plates and transformation was carried out according to Dörnte and Kües (2012). Submerged cultivation of positive transformants was conducted in modified Kjalke (mKjalke) medium (Rühl et al. 2013) supplemented with 0.1 mM CuSO_4_.

### Gene amplification

Total gDNA of *C.* *cinerea* strain FA2222 was isolated from fungal mycelia according to Liu et al. (2000). For amplification of the *lcc8* gene (Hoegger et al. 2004; gene accession number AY338764) via PCR from gDNA, a primer pair was used with homologous overlaps to the DNA regions of the *gpdII*-promotor and *lcc1*-terminator present in the pYSK7 plasmid (Kilaru et al. 2006a, b): pYMS33_for (5′-TTCTGTCGATACCATGACCCTCACCAACGCAAACTTGTCGCCGGATGGCT-3′) and pYMS33_rev (5′-GCCCTCTGGTCAACTATAATATTATTCACGACGAATCAGTAGCCGCAGCA-3′). PCR was performed using the Phusion^®^ High-Fidelity DNA Polymerase Kit (New England Biolabs, Frankfurt, Germany) according to the instruction manual. pYSK7 was linearized by restriction digestion using the enzymes FD-*Hpa*I and FD-*Bam*HI (ThermoScientific, Schwerte, Germany) according to manufacturer´s instructions. The PCR products and the linear fragments derived from pYSK7 were detected via agarose gel electrophoresis and purified from gel pieces using the NucleoSpin PCR cleanup Kit (Macherey-Nagel, Düren, Germany). Yeast strain RH1385 was transformed with the PCR product of pYMS33_for/rev and the linearized pYSK7 for homologous recombination as described elsewhere (Galperin et al. 2016) to generate the final plasmid pYMS33. Clones were analyzed by colony PCR using pYSK7 expression cassette specific primers (*gpdII*_for 5′-CCATCTCCGTTTTCTCCCATC-3′; *lcc1*-term_rev 5′-CTGGCCCTCTGGTCAACTATAAT-3′).

### Plasmid DNA isolation from *S. cerevisiae* RH1385

Positive yeast clones, proofed by colony PCR, were used for plasmid DNA isolation. A 10 mL YSDO overnight culture of positive clones was spun down for 5 min at 500*g* to sediment cells. Supernatant was discarded and cell pellet was washed in 0.5 mL ddH_2_O. Cells were transferred to 1.5 mL Eppendorf tube and spun down again for 5 s at 18,000*g*. Supernatant was discarded and the cell pellet was dissolved in residual liquid. 200 µL yeast lysis buffer (4 mL Trition x-100, 20 mL 10% SDS, 4 mL 5 M NaCl, 400 µL 0.5 M EDTA, 2 mL 1 M Tris pH 8.0, filled up to 200 mL with ddH_2_O), 200 µL phenol:chloroform:isoamylalcohol (25:24:1) and 0.3 g acid washed glass beads (425–600 µm) were added to the yeast cells in this sequence. The cells were vortexed for 10 min and 200 µL TE-buffer (pH 8.0) was added. After 5 min centrifugation, 400 µL of the aqueous phase was transferred to a new tube. 40 µL 3 M sodium acetate (pH 5.5) and 1 mL of 96% ethanol were added and mixed. The preparation was placed for 15 min in − 20 °C and afterwards spun down for 20 min. Supernatant was discarded and 400 µL TE buffer and 4 µL RNase A (10 mg mL^−1^) were added to the nucleic acid pellet. RNA was digested for 15 min at 37 °C. 10 µL of 4 M ammonium acetate and 1 mL of 96% ethanol were added and mixed again. The nucleic acids were spun down again for 2 min and supernatant was discarded. The DNA-pellet was washed with 500 µL 70% ethanol for 5 min. After removing ethanol and drying the pellet, the DNA was dissolved in 50 µL ddH_2_O. Isolated plasmid DNA was used for electro-transformation of *E.* *coli* JM109 with the Eppendorf Eporator^®^ (Eppendorf, Hamburg, Germany). Plasmid pYMS33 was extracted from *E. coli* and sequenced by SeqLab (Göttingen, Germany). *C.* *cinerea* strain FA2222 was co-transformed according to the transformation protocol of Dörnte and Kües (2012) with 1–2 µg of the marker plasmid pCc1001 complementing the tryptophan auxotrophy of FA2222 and with the same amount of pYMS33. Additionally, pCc1001 was transformed solely into FA2222 to obtain a control transformant. Picked clones were grown on minimal media agar plates supplemented with 0.5 mM 2,2′-azino-bis-(3-ethylbenzthiazoline-6-sulfonate) diammonium salt (ABTS; AppliChem GmbH, Darmstadt, Germany) until a faint coloring appeared. Transformants which were positive in the ABTS plate assay were set on minimal media plates and cultured for 3–4 days. When there was enough mycelia a fast microwave based DNA extraction was performed according to the method of Dörnte and Kües (2013) to verify successful integration of the recombinant *lcc8* expression cassette into the *C.* *cinerea* genome by means of colony PCR using the pYSK7 expression cassette specific primers (*gpdII*_for 5′-CCATCTCCGTTTTCTCCCATC-3′; lcc1-term_rev 5′-CTGGCCCTCTGGTCAACTATAAT-3′).

### Laccase purification

At the day of highest laccase activity, the supernatant of pYMS33 clone 12 culture was harvested and filtered by folded filter MN616^1/4^ Ø270 mm (Macherey-Nagel, Düren, Germany). The pH of supernatant was adjusted to pH 6.4 followed by centrifugation at 4700*g* at 4 °C for 30 min and filtered again. Before using a FPLC-column the supernatant was filtered with an Ultrasette™ Screen Channel TFF Device (Pall, Dreieich, Germany) to exclude all particles bigger than 300 kDa. All FPLC purification steps were performed with columns of GE Healthcare (Freiburg, Germany) and using the BioLogic DuoFlow system of Bio-Rad (Düsseldorf, Germany). The ultra-filtrated supernatant was applied to DEAE-FF anion exchange (AEX) XK26 column, using 20 mM KH_2_PO_4_ (pH 6.4) as loading buffer and 20 mM KH_2_PO_4_ + 1 M NaCl as elution buffer. Collected fractions were screened for laccase activity by ABTS assay and active fractions were pooled. Pooled fractions were filtered using the VivaFlow 200 with a 10 kDa membrane (Sartorius, Göttingen, Germany) and thereby were resalted with loading buffer 20 mM KH_2_PO_4_ + 1 M (NH_4_)_2_SO_4_ for hydrophobic interaction chromatography (HIC). A HiPrep16/10 highsub column with Phenyl FF as solid phase was used for the HIC. HIC fractions were eluted with 20 mM KH_2_PO_4_. Collected fractions were screened again for laccase activity, pooled and desalted.

### Laccase activity assay

Laccase activity was determined in 96-well plates at room temperature with 10 mM ABTS in 150 mM sodium acetate buffer at pH 5.0 as described earlier by Rühl et al. (2013). Oxidation of ABTS into its cation radical (ABTS·^+^) was measured by an increase of absorbance at 420 nm (ε = 36.000 M^−1^ cm^−1^) for 10 min (Johannes and Majcherczyk 2000). One unit of activity was defined as the amount of enzyme needed to oxidize 1 µmol ABTS per min and activities are given in U per volume.

### Enzyme analysis

#### Electrophoresis and staining

For separation of proteins and detection of laccases, samples were applied to SDS-PAGE according to Laemmli (1970) with 4% stacking and 12% resolving polyacrylamide gels. Laccase zymogram was performed according to Rühl et al. (2013). Silver staining of an SDS gel was performed according to Blum et al. (1987), (Table 1). Colloidal Coomassie staining was performed according to Dyballa und Metzger (2009).

**Table 1:** Purification of Lcc8 from culture supernatant

Purification step	Total laccase activity [U]	Total protein amount [mg]	Specific activity [U/mg]	Yield [%]	Purification factor
Supernatant	133	28.6	4.7	100	1.0
Ultrafiltration	127	22.7	5.6	95	1.2
AEX	85	5.4	15.7	64	3.3
HIC	25	0.5	46.3	19	9.9

#### Isoelectric focusing

For determination of the pI values the SERVAGel™ IEF 3–10 system was used according to manufacturer’s manual. 20 mU of purified laccase for native staining were used. The SERVA IEF Marker 3–10 liquid mix was used for determination of the pI values. Native staining was performed according to Rühl et al. (2013).

#### Protein identification

Protein bands of the Coomassie- or native stained gels corresponding to laccase activity were cut with a razor blade and transferred to 1.5 mL LoBind tubes (Eppendorf, Hamburg, Germany). Gel pieces were washed and proteins digested as previously described (Gundry et al. 2009). Briefly, gels were washed with 50% methanol (0.1% formic acid), destained with 50 mM ammonium bicarbonate in 50% acetonitrile, washed with water and dehydrated with acetonitrile. Proteins were reduced with 1,4-dithiothreitol and alkylated with iodoacetamide. Finally, proteins were digested in-gel by trypsin at 37 °C overnight. Extracted peptides were further purified on self-made C18-STAGE tips (Rappsilber et al. 2007). Peptides were analyzed by nanoLC (Eksigent 425, Sciex) coupled to TripleTOF 5600+ mass spectrometer (Sciex, Darmstadt, Germany) and raw MS-data were processed using ProteinPilot (Sciex). The protein fasta-file of *C. cinerea* AmutBmut pab1-1 (v1.0) used for data searches was downloaded (https://genome.jgi.doe.gov/Copci_AmutBmut1/Copci_AmutBmut1.home.html) from JGI (DOE, Walnut Creek, CA, USA).

#### Biochemical characterization

Besides ABTS some other substrates were used for determination of laccase activity, which were 2,6-dimethoxyphenol (DMP), 2-methoxyphenol (guaiacol) and syringaldazine (SGZ, 4-hydroxy-3,5-dimethoxybenzaldehyde azine). The increase of extinction was observed spectroscopically at 420 nm (ε = 36.000 M^−1^ cm^−1^), 468 nm (ε = 49.600 M^−1^ cm^−1^), 436 nm (ε = 6.400 M^−1^ cm^−1^) and 526 nm (ε = 65.000 M^−1^ cm^−1^) for ABTS, DMP, guaiacol and syringaldazine, respectively (Matsumura et al. 1986; Wariishi et al. 1992; Slomczynski et al. 1995; Eggert et al. 1996). One unit of enzyme activity was defined as the amount of enzyme oxidizing 1 μmol substrate per min. For determination of the optimal pH-value Britton–Robinson universal buffer pH 2–12 was used. The pH stability was determined by incubating 150 mU laccase in 1 mL universal buffer (pH 2–12) for up to 24 h. At defined intervals, samples were collected and laccase activity was measured with ABTS in 150 mM sodium acetate buffer (pH 5.0). For the determination of the optimal temperature, the laccase activity was measured at the temperatures ranging from 11 to 71 °C with ABTS in 150 mM sodium acetate buffer (pH 5.0) using cuvettes. The reaction temperature has been measured directly in the cuvettes resulting in the distinct values of 11 °C, 20 °C, 28 °C, 36 °C, 45 °C, 54 °C, 63 °C and 71 °C. The thermal stability was determined by incubation of the laccase in universal buffer pH 8 for at least 5 h at 10 °C, 30 °C, 50 °C and 70 °C. The activity was measured with ABTS in 150 mM sodium acetate buffer (pH 5.0).

Deglycosylation of Lcc8 was performed with PNGase F according to the manufacturer protocol (New England Biolabs, Frankfurt, Germany) and monitored by SDS-PAGE (see above).

## Results

**Fig. 1:**
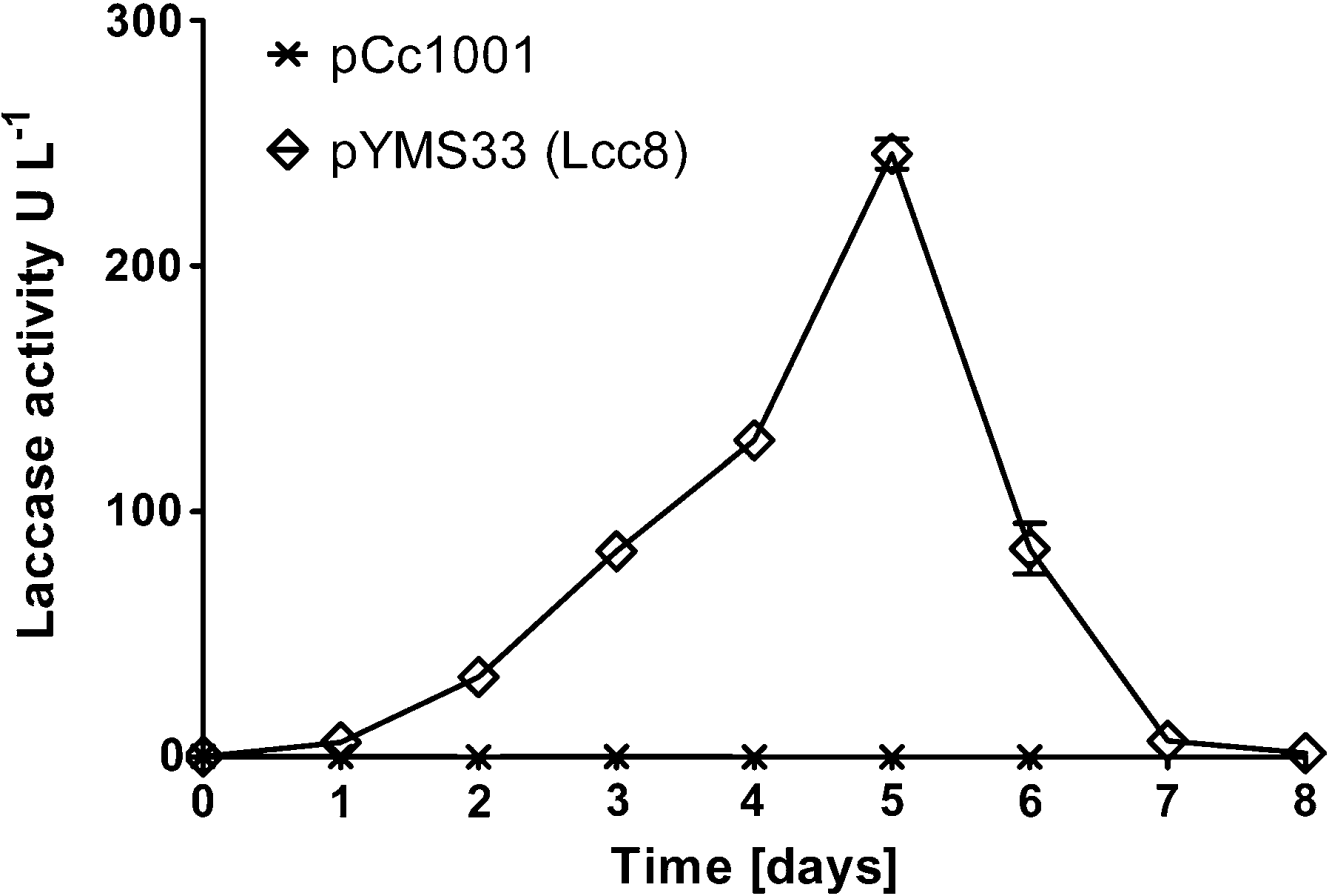
Laccase activity of pYMS33-12 and pCc1001 cultures. The laccase activity was monitored by ABTS assay

To express laccase Lcc8 homologously in *C. cinerea*, the most suitable plasmid pYSK7 constructed by Kilaru et al. (2006b) for the expression of Lcc1 was modified in this study. The *lcc8* construct was introduced into the linearized pYSK7 plasmid via homologus recombination in the yeast *Saccharomyces cerevisiae* RH1385. The resulting plasmid pYMS33 comprised of the *lcc8* gene under control of the glyceraldehyde 3-phosphate dehydrogenase II (*gpdII*) promotor from *Agaricus bisporus* and the *lcc1*-terminator of *C. cinerea* laccase lcc1 both revealed to be functional in *C. cinerea* laccase expression (Kilaru et al. 2006b). Two transformations of *C.* *cinerea* strain FA2222 with pYMS33 (Additional file 1: Figure S1) resulted in a total number of 80 transformants. All pYMS33 transformants as well as four pCc1001 control transformants were cultivated on ABTS supplemented agar plates for up to 3 days at 37 °C. For almost all transformants a very faint coloration was observed, whereas one pYMS33 transformant (pYMS33-12) showed a distinct green halo. PCR of pYMS33-12 genomic DNA using primers *gpdII*_for and *lcc1*-term_rev confirmed the presence of the *lcc8* expression cassette in pYMS33-12. Submerged cultivation of pYMS33-12 in mKjalke medium supplemented with 0.1 mM CuSO_4_ resulted in increasing laccase production in the supernatant with a maximum activity of 250 U L^−1^ at day 5 of cultivation (Fig. 1). With ongoing cultivation, pYMS33-12 showed a rapid decrease in laccase activity until the end of cultivation. In parallel cultures of the control transformant pCc1001, no laccase activity was observed during the whole cultivation.

The supernatant of a subsequent cultivation of pYMS33-12 in the same medium was used to purify Lcc8. Consecutive purification steps (ultrafiltration, anion exchange chromatography—AEX, and hydrophobic interaction chromatography—HIC) resulted in a final purification factor of 10, which accounts for a specific laccase activity of 46.3 U mg^−1^ (Table 1). Thus, around one-fifth (19%) of the laccase activity from the supernatant remained in the purified sample.

**Fig. 2:**
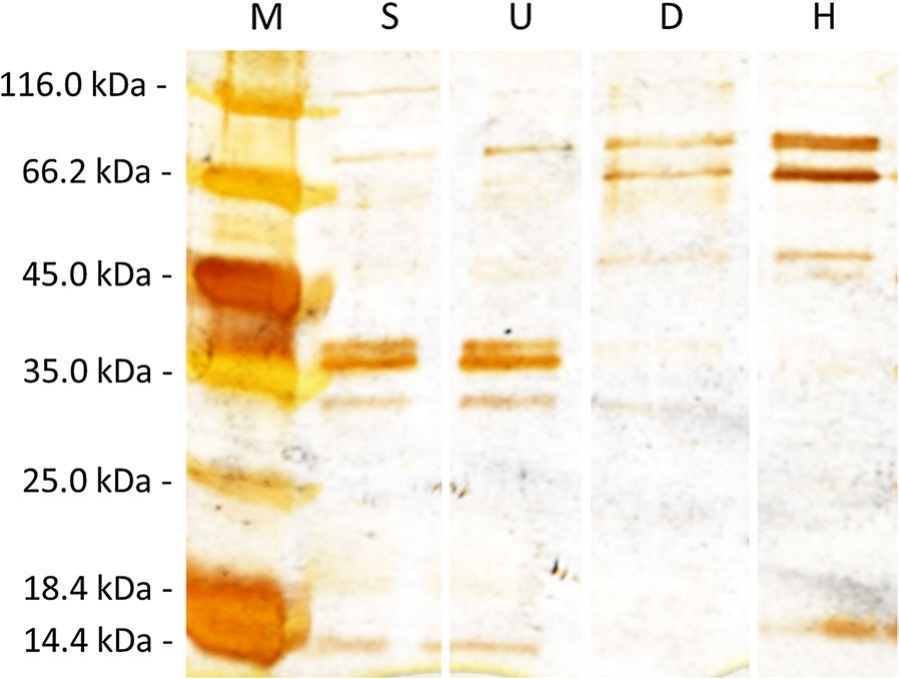
SDS-PAGE of Lcc8 purification steps stained with silver under denaturizing conditions. *M* Pierce™ unstained protein MW Marker, *S* supernatant, *U* ultrafiltration, *D* AEX (DEAE-Sepharose) and *H* HIC

**Fig. 3:**
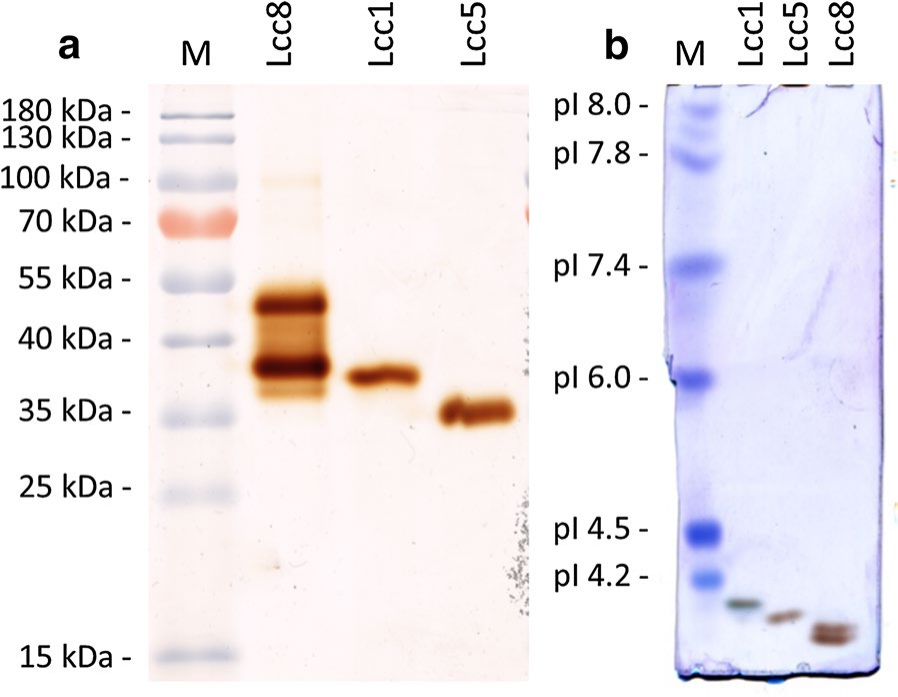
**a** Zymogram of purified Lcc8, Lcc1 and Lcc5 (all 20 mU) and stained with laccase substrates 10 mM MBTH and 10 mM DHPPA. **b** Isoelectric focusing PAGE stained with laccase substrate MBTH/DHPPA. The figure shows the samples M = SERVA IEF Marker 3–10 Liquid Mix, purified Lcc1, Lcc5 and Lcc8

SDS-PAGE reveals increasing band intensity for two bands with the sizes 64 kDa and 77 kDa correlating with the grade of purification (Fig. 2). In addition, a smaller band of around 50 kDa is present in the HIC fraction. A laccase zymogram of the HIC fraction stained with the substrate mixture 3-methyl-2-benzothiazolinone-hydrazone hydrochloride (MBTH) and 3,4-dihydroxyhydrocinnamic acid (DHPPA), which is known to be highly efficient for laccase activity staining (Rühl et al. 2013), shows two main laccase bands for the purified pYMS33-12 fraction (Fig. 3a). Furthermore, the laccase activity bands for Lcc8 showed a different running pattern in the SDS-PAGE in correlation to the control laccases Lcc1 and Lcc5 both showing only one main laccase band.

The two putative Lcc8 laccase bands (Fig. 3a) of a zymogram were cut and analyzed by nano-LC–MS/MS. The proteomic analysis revealed that both analyzed bands belong to the *C.* *cinerea* laccase Lcc8 (Additional file 1: Table S1).

### Laccase characterization

**Fig. 4:**
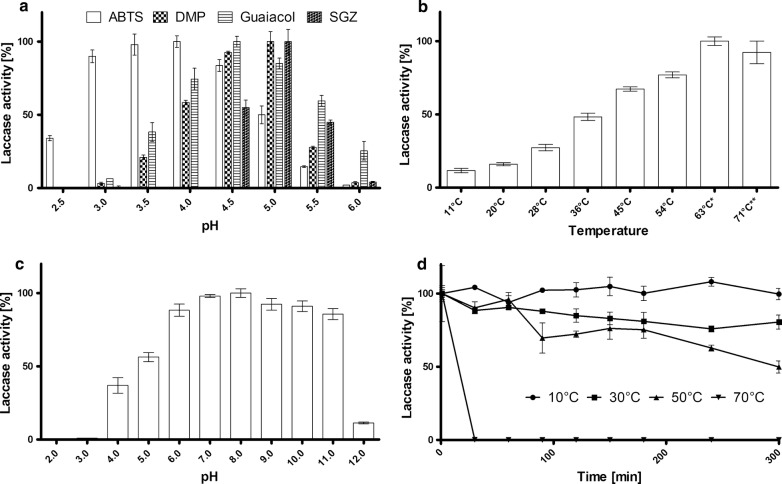
Characterization of Lcc8. **a** Detection of substrate specificity and pH optimum. **b** Detection of optimal reaction temperature with ABTS. *The activity was stable for 5 min and then began to decrease slowly. **The activity was stable for 3 min and then began to decrease rapidly. **c** Analysis of optimal storage pH. **d** Enzyme stability at different temperatures

For determination of pI-values an isoelectric focusing (IEF) was performed and proteins were stained with the laccase specific substrate mixture MBTH and DHPPA. The IEF (Fig. 3b) shows two active laccase bands in the Lcc8 fraction with a lower pI than the control laccases Lcc1 and Lcc5. Extrapolation of marker pIs led to pI-values of about 3.3 and 3.4, respectively. Activity assays with four different substrates in the pH range from pH 2 to pH 12 showed highest enzyme activities between pH 3.5 and pH 5.0 and no laccase activity could be detected below a pH of 2.5 or above 6.0. Lcc8 showed optimal conditions for the oxidation of ABTS at a pH of 3.5–4.0, DMP at a pH 5.0, guaiacol at a pH of 4.5 and SGZ at a pH of 5.0 (Fig. 4a). The optimal temperature for Lcc8 using ABTS as a substrate was 63 °C, although at this temperature the activity was stable only for 5 min and then decreased slowly. At temperatures of 54 °C and below, no decrease in activity was observed during measurement. Higher temperatures than 63 °C led to a quick deactivation of Lcc8 and resulted in decreased laccase activity (Fig. 4b).

Testing of storage stability of Lcc8 in universal buffer at different pH values showed that Lcc8 is stable in an alkaline pH-range. Surprisingly, at least 50% of the enzyme activity remained after 48 h in buffers from pH 6 to pH 11, with highest stability at around pH 8 (Fig. 4c). Accordingly, Lcc8 was mixed with universal buffer pH 8 to reveal the temperature stability (Fig. 4d). After 30 min at a temperature of 70 °C, Lcc8 showed no activity. This correlates with the results of the determination of the optimal reaction temperature, where already after 3 min at 71 °C the activity decreased rapidly. At a temperature of 50 °C, Lcc8 was more stable, remaining 50% of its activity after 300 min. Lower temperatures showed minimal (30 °C) or no (10 °C) activity loss over a tested period of 300 min at pH 8.

## Discussion

Laccases of *C. cinerea* cluster into two distinct groups, the larger laccase sensu stricto subfamily 1 and the smaller subfamily 2 (Kües and Rühl 2011). Within subfamiliy 1, Lcc8 is outlined from the other *C.* *cinerea* laccases (Kilaru et al. 2006a) assuming a special role in *C. cinerea*. In a previous work, Kilaru et al. (2006a) was not successful in homologous expression of Lcc8. Neither the native *lcc8* sequence nor the “long *lcc8*” sequence, which started 412 bp upstream of the predicted start codon, resulted in an active laccase. The SignalP 4.1 algorithm (Petersen et al. 2011) predicted no signal sequence for the Lcc8 amino acid sequence (accession number: BK004118), but one signal sequence of 23 amino acid length for the long Lcc8 (accession number: A8N4I7). The missing signal peptide in Lcc8 and the yet not detected Lcc8 peptides in analyzed *C. cinerea* wild type cultures (Rühl et al. 2013; Hu et al. 2018) led to the assumption that Lcc8 might not be transported outside of the fungal cell and, thus, acting intracellularly. By exchanging the N-terminal sequence of the Lcc8 with the signal peptide of Lcc1 (Additional file 1: Figures S1 and S2), known to be constantly expressed, a successful expression of Lcc8 was possible. Although in contrast to previous studies on recombinant enzyme production in *C.* *cinerea* (Kilaru et al. 2006b; Galperin et al. 2016), co-transformation experiments in this work resulted only in one distinct positive clone out of 80 tested clones accounting for a poor co-transformation efficiency of only 1.25%. Generally, efficiencies of more than 40% and 50% are possible as we could show in earlier co-transformations with *C.* *cinerea* using pCc1001 as marker plasmid (Kilaru et al. 2006a, b; Galperin et al. 2016). The poor transformation efficiency for Lcc8 expression can be due to an insufficient plasmid DNA concentration, which is very important for high transformation efficiencies. For a co-transformation of *C. cinerea*, Dörnte and Kües (2016) showed that lowering the amount of expression plasmid, while applying a constant concentration of marker plasmid pCc1001, lead to a reduced number of clones and to a low co-transformation efficiency of only 5–10%. Another explanation might be that positive clones have been overlooked due to their low laccase activity resulting in no observable oxidation of ABTS during cultivation on agar plates for up to 3 days. Nevertheless, PCR and peptide analysis confirmed the integration of the expression cassette and the expressed laccase to be Lcc8.

The positive transformant pYMS33-12 was cultured in liquid medium under conditions where native laccases are not produced (Kilaru et al. 2006a, b; Rühl et al. 2013). This was confirmed by the cultivation of the control transformant pCc1001 under the same conditions showing any laccase activity during submerged fermentation over a period of 8 days (Fig. 1). The obtained maximal laccase activity of around 0.25 U mL^−1^ in pYMS33-12 cultures is ten times lower than in comparable cultures of the *C.* *cinerea* transformant pYSK7 producing the *C. cinerea* laccase Lcc1. Kilaru et al. (2006b) demonstrated that the pYSK7 transformant cultivated in YMG (yeast, malt extract, glucose) medium supplemented with 0.1 mM CuSO_4_ showed a maximal activity of 3 U mL^−1^. This activity was dramatically higher than in cultures without the addition of 0.1 mM CuSO_4_ (Kilaru et al. 2006b). A similar activity of 2.9 U mL^−1^ was achieved when pYSK7 was cultivated in mKjalke medium supplemented with 0.1 mM CuSO_4_ at 37 °C (Rühl et al. 2018). Laccases are multi-copper oxidases, which require four copper atoms per active laccase molecule (Messerschmidt 1997). Thus, addition of CuSO_4_ is generally applied to yield high amounts of active enzymes (Larrondo et al. 2003; Kilaru et al. 2006b). The protein yield of Lcc8 calculated on basis of its specific activity (46.3 U mg^−1^) and volumetric activity (250 U L^−1^) was around 5 mg L^−1^. This is in the same range as laccase yields in yeast hosts, such as *Pleurotus ostreatus* laccases in *Kluyveromyces lactis* with around 2 mg L^−1^ or *Ganoderma lucidum* laccase in *Pichia pastoris* with 6 mg L^−1^ (Piscitelli et al. 2005; You et al. 2014). Although yields of Lcc8 are far below recombinant laccases in filamentous ascomycetous host (see Mate and Alcalde 2015), pYMS33-12 produced sufficient amounts for characterization of Lcc8.

SDS-PAGE analysis showed two bands of 64 kDa and 77 kDa for Lcc8, which are larger than the theoretical molecular weight of 59.7 kDa calculated from AA-sequence. Other *C. cinerea* laccases showed a smaller molecular weight in SDS-PAGE analyses ranging from 54 to 66 kDa for Lcc1, Lcc2 and Lcc6 (Yaver et al. 1999; Tian et al. 2014; Wang et al. 2014). The two bands in the SDS-PAGE (Fig. 2) correspond to the presence of two bands in the zymogram (Fig. 3a), both being confirmed as Lcc8 bands by LC–MS. In addition, deglycosylation experiment of purified Lcc8 with PNGase F did not result in a single band. Thus, the hypothesis of a glycosylation impact on the molecular weight difference as shown by several previous works on recombinant laccases (Otterbein et al. 2000; Sigoillot et al. 2004; Madzak et al. 2005) can be discarded. Also a missing cleavage of the Lcc1 signal peptide, which has a calculated molecular weight of less than 3 kDa, could not led to a molecular weight difference of 13 kD. Intron retention during splicing would also result in a larger molecule. However, in the *lcc8* open reading frame of both sequenced *C. cinerea* genomes (AmutBmut: fgenesh1_kg.162_#_19_#_Locus292v1rpkm600.11, Okayama 7: CC1G_05965T0) only one intron could be retained without facing a frame shift. This retention would lead to an Lcc8 sequence with an additional 19 amino acids accounting for around 2 kDa. Thus, the existence of two Lcc8 bands of similar intensity cannot be clarified yet.

Lcc8 showed similar characteristics to the other *C. cinerea* laccases Lcc1, Lcc2, Lcc6 and Lcc9 (Table 2); (Schneider et al. 1999; Yaver et al. 1999; Pan et al. 2014; Tian et al. 2014; Wang et al. 2014). The optimal pH of 4.0 for ABTS is similar for Lcc8 and Lcc1 (Schneider et al. 1999), while the pH optimum for all other analyzed *C. cinerea* laccases is lower. Lcc2 and Lcc6 show an optimal ABTS oxidation of ABTS at pH 3 and Lcc9 at pH 2.5 (Table 2). For the phenolic substrates SGZ, DMP and SGZ, Lcc8 shows a lower pH optimum than Lcc1 and Lcc9 (Schneider et al. 1999; Pan et al. 2014). Generally, in comparison to all other so far characterized *C. cinerea* laccases, Lcc8 has a narrower optimal pH for the different tested substrates. The optimal range for pH stability for Lcc8 (pH 5–11) is smaller than the range for Lcc2 (pH 2–11) (Tian et al. 2014), but larger than for Lcc6 (pH 3–6) (Wang et al. 2014). Nevertheless, the stability of Lcc2 was tested at 4 °C, whereas Lcc8 showed very good stability at 24 °C for 24 h. Even after 48 h at 24 °C, Lcc8 retained 50% of its initial activity between pH 6–11.

**Table 2:** Comparison of properties of different *C. cinerea* laccases

Enzyme properties	Substrate	Lcc1 (Schneider et al. 1999)	Lcc2 (Tian et al. 2014)	Lcc6 (Wang et al. 2014)	Lcc9 (Pan et al. 2014)	Lcc8 this study
Molecular weight [kDa]		66.0	54.0	57.4	n. d.	64 and 77
Optimal pH	ABTS	4.0	2.6	3.0	2.5	4.0
	DMP	n. d.	n. d.	n. d.	6.5	5.0
	guaiacol	n. d.	n. d.	n. d.	6.5	4.5
	SGZ	6.5	n. d.	n. d.	6.5	5.0
Thermal stability (50 °C)		> 200 min	ca.50 min	> 60 min	n. d.	300 min
Optimal temperature		60–70 °C	45 °C	40 °C	60 °C	54–63 °C
pH stability (≥ 50%)		7.0–10.0 4 °C, 14 days	2.0–11.0 4 °C, 24 h	2.5–6.0 4 °C, 24 h	4.5–6.5 37 °C, 8 h	5.0–11.0 24 °C, 24 h
pI value		3.5	n. d.	n. d.	n. d.	3.3/3.4

The optimal temperature for Lcc8 is comparable to those of Lcc1 and Lcc9 (Schneider et al. 1999; Pan et al. 2014) all showing highest activity against ABTS at around 60–70 °C. Lcc2 was shown to have a broad temperature range with a maximum at 45 °C (Tian et al. 2014) and Lcc6 had a maximum at 40 °C (Wang et al. 2014). Outstanding was the thermal stability of Lcc8 at 50 °C, which was higher than for other characterized *C. cinerea* laccases (Table 2). While activity of Lcc2 (Tian et al. 2014) decreased after 60 min to less than 50%, Lcc8 showed only small decrease after 60 min and retained 50% of its activity after 300 min. Lcc6 (Wang et al. 2014) was only incubated for 60 min and, as well as Lcc8, showed only a slight reduction in laccase activity at 50 °C.

Although similar to the already published *C. cinerea* laccase characteristics, Lcc8 has specific features such as a very narrow optimal pH range for different substrates as well as a high stability at alkaline pH. This specificity confirm the differences between the *C. cinerea* laccases as shown previously in phylogenetic analyses (Hoegger et al. 2004, 2006; Kües and Rühl 2011). The introduced technique of N-terminal signal peptide substitution in *C. cinerea* can help to produce other laccases not secreted by wildtype *C.* *cinerea* strains or even by other fungi of the phylum Basidiomycota.

## Additional file

**Additional file 1: Table S1.** Scores of laccase peptides detected by LC-MS/MS using the ProteinPilot software. **Figure S1.** A Plasmid map of pYMS33 used for transformation the *C.* *cinerea* laccase *lcc8* based on the pYSK7 plasmid of Kilaru et al. (2006b). Instead of the native signal peptide of *lcc8* the signal peptide of *lcc1* was used. The constitutive glyceraldehyde 3-phosphate dehydrogenase II (*gpdII*) promotor promotes expression of *lcc8* and the *lcc1*-terminator stops transcription. *URA3* encodes a uracil synthase important for selection of RH1385 clones by auxotrophy and *ampR* leads to resistance to ampicillin in *E.* *coli*. 2Âµm ori and ColE ori are responsible for replication of plasmid in yeast and *E.* *coli*. B Deduced amino acid sequence of Lcc8. The underlined letters are representing the Lcc1 signal peptide. The * marks the signal peptide cleavage side predicted by SignalP 4.1 followed by twelve additional amino acids of the Lcc1 sequence as a linker. The histidine and cysteine highlighted in red boxes are involved in copper binding according to Kilaru et al. (2006a). Putative N-glycosylation sites are highlighted in green boxes. **Figure S2.** Alignment of *C. cinerea* deduced amino acid sequences of long Lcc8 (Cci_longLcc8, Accession number: A8N4I7), Lcc8 (Cci_Lcc8, Accession number: BK004118) and Lcc8 with the Lcc1 signal peptide (Cci_Lcc1SP_Lcc8).

## Abbreviations

ABTS: 2,2′-azino-bis(3-ethylbenzothiazoline-6-sulfonic acid) diammonium salt
AEX: anion exchange chromatography
DHPPA: 3,4-dihydroxyhydrocinnamic acid
HIC: hydrophobic interaction chromatography
IEF: isoelectric focusing
MBTH: 3-methyl-2-benzothiazolinone-hydrazone hydrochloride
LC–MS: liquid chromatography–ESI coupled to mass spectrometer
