# Supplementary material for: Signal peptide replacement resulted in recombinant homologous expression of laccase Lcc8 in Coprinopsis cinerea
Source: AMB Express. 2019 Mar 15;9:36. doi: 10.1186/s13568-019-0761-1 (PMC6420550; doi:10.1186/s13568-019-0761-1)
Supplement: Supplementary file 1 — Additional file 1: Table S1. Scores of laccase peptides detected by LC-MS/MS using the ProteinPilot software. Figure S1. A Plasmid map of pYMS33 used for transformation the C. cinerea laccase lcc8 based on the pYSK7 plasmid of Kilaru et al. (2006b). Instead of the native signal peptide of lcc8 the signal peptide of lcc1 was used. The constitutive glyceraldehyde 3-phosphate dehydrogenase II (gpdII) promotor promotes expression of lcc8 and the lcc1-terminator stops transcription. URA3 encodes a uracil synthase important for selection of RH1385 clones by auxotrophy and ampR leads to resistance to ampicillin in E. coli. 2Âµm ori and ColE ori are responsible for replication of plasmid in yeast and E. coli. B Deduced amino acid sequence of Lcc8. The underlined letters are representing the Lcc1 signal peptide. The * marks the signal peptide cleavage side predicted by SignalP 4.1 followed by twelve additional amino acids of the Lcc1 sequence as a linker. The histidine and cysteine highlighted in red boxes are involved in copper binding according to Kilaru et al. (2006a). Putative N-glycosylation sites are highlighted in green boxes. Figure S2. Alignment of C. cinerea deduced amino acid sequences of long Lcc8 (Cci_longLcc8, Accession number: A8N4I7), Lcc8 (Cci_Lcc8, Accession number: BK004118) and Lcc8 with the Lcc1 signal peptide (Cci_Lcc1SP_Lcc8). [file 13568_2019_761_MOESM1_ESM.docx]

**Additional Material**

**Table S1** Scores of laccase peptides detected by LC-MS/MS using the ProteinPilot software

| **Lcc8 (Q08AB9)peptides*** | **Upper band** | **Lower band** |
| --- | --- | --- |
| R.TQYCDGLR.G |  | 12 |
| R.GPLVIYDPEDPYR.D | 17 | 19 |
| R.YVGGPLVDLAIINVVPGK.R | 22 |  |
| R.YVGGPLVDLAIINVVPGKR.Y |  | 26 |
| R.YSAILTADQPIGNYWIR.A |  | 17 |
| R.ALPNSGNNNLFTGFIDGTNSAILR.Y | 21 |  |
| K.SAGIDTPYNYVNPVK.R | 16 | 16 |
| K.SAGIDTPYNYVNPVKR.D | 22 | 19 |
| R.DVVSTGDVGSNTTIR.F |  | 17 |

*only peptides with the a confidence of ≥99% are shown


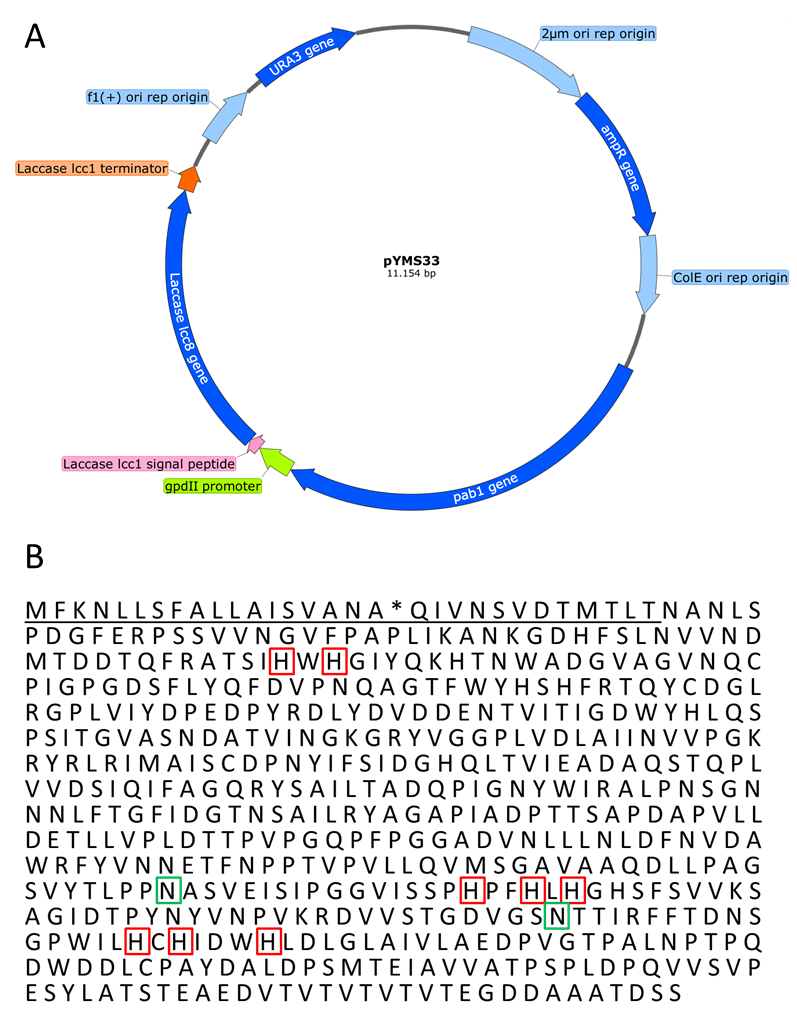


Figure S1 A Plasmid map of pYMS33 used for transformation the *C. cinerea* laccase *lcc8* based on the pYSK7 plasmid of Kilaru et al (2006b). Instead of the native signal peptide of *lcc8* the signal peptide of *lcc1* was used. The constitutive glyceraldehyde 3-phosphate dehydrogenase II (*gpdII*) promotor promotes expression of *lcc8* and the *lcc1*-terminator stops transcription. *URA3* encodes a uracil synthase important for selection of RH1385 clones by auxotrophy and *ampR* leads to resistance to ampicillin in *E. coli*. 2Âµm ori and ColE ori are responsible for replication of plasmid in yeast and *E. coli*. B Deduced amino acid sequence of Lcc8. The underlined letters are representing the Lcc1 signal peptide. The * marks the signal peptide cleavage side predicted by SignalP 4.1 followed by twelve additional amino acids of the Lcc1 sequence as a linker. The histidine and cysteine highlighted in red boxes are involved in copper binding according to Kilaru et al. (2006a). Putative N-glycosylation sites are highlighted in green boxes.


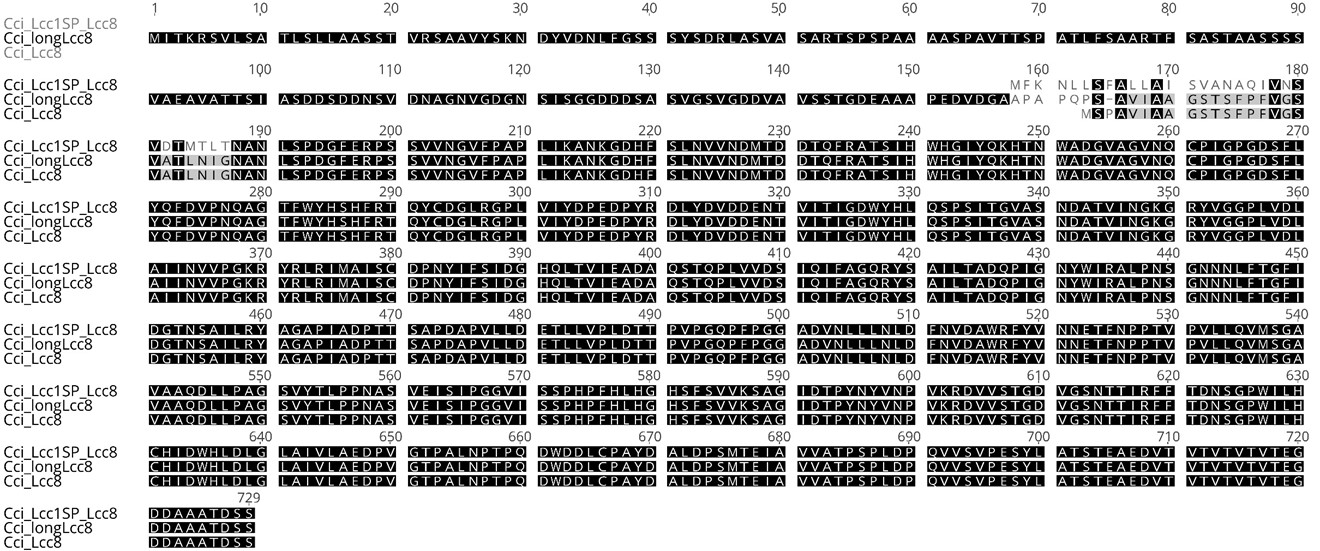


Figure S2 Alignment of *C. cinerea* deduced amino acid sequences of long Lcc8 (Cci_longLcc8, Accession number: A8N4I7), Lcc8 (Cci__Lcc8, Accession number: BK004118) and Lcc8 with the Lcc1 signal peptide (Cci_Lcc1SP_Lcc8).
